# *MECP2*, a gene associated with Rett syndrome in humans, shows conserved coding regions, independent *Alu* insertions, and a novel transcript across primate evolution

**DOI:** 10.1186/s12863-015-0240-x

**Published:** 2015-07-07

**Authors:** Maria Carolina Viana, Albert Nobre Menezes, Miguel Angelo M. Moreira, Alcides Pissinatti, Héctor N. Seuánez

**Affiliations:** Genetics Division, Instituto Nacional de Câncer, Rua André Cavalcanti 37, 4th floor, 20231-050 Rio de Janeiro, RJ Brazil; Centro de Primatologia do Rio de Janeiro, Rio de Janeiro, Brazil; Department of Genetics, Universidade Federal do Rio de Janeiro, Rio de Janeiro, Brazil

**Keywords:** *MECP2* gene, Primates, *Alu* inserts, Novel transcript

## Abstract

**Background:**

The methyl-CpG Binding Protein two gene (*MECP2*) encodes a multifunctional protein comprising two isoforms involved in nuclear organization and regulation of splicing and mRNA template activity. This gene is normally expressed in all tissues, with a higher expression level in the brain during neuronal maturation. Loss of *MECP2* function is the primary cause of Rett syndrome (RTT) in humans, a dominant, X-linked disorder dramatically affecting neural and motor development.

**Results:**

We investigated the molecular evolution of *MECP2* in several primate taxa including 36 species in 16 genera of neotropical (platyrrhine) primates. The coding region of the *MECP2_e2* isoform showed a high level of evolutionary conservation among humans and other primates, with amino acid substitutions in 14 codons and one in-frame insertion of a single serine codon, between codons 357 and 358, in *Ateles paniscus*. Most substitutions occurred in noncritical regions of MECP2 and the majority of the algorithms used for analyzing selection did not provide evidence of positive selection. Conversely, we found 48 sites under negative selection in different regions, 23 of which were consistently found by three different algorithms. Similar to an inverted *Alu* insert found previously in a lesser ape at a parallel location, one *Alu* insertion of approximately 300 bp in *Cebus* and *Sapajus* was found in intron 3. Phylogenetic reconstruction of the intron 3 data provided a topology that was coincident with the consensus arrangement of the primate taxa. RNAseq data in the neotropical primate *Callimico goeldii* revealed a novel transcript consisting of a noncontinuous region of the human-homologous intron 2 in this species; this transcript accounted for two putative polypeptides.

**Conclusions:**

Despite the remarkable evolutionary conservation of *MECP2*, one in-frame codon insertion was observed in *A. paniscus*, and one region of intron 3 was affected by a trans-specific *Alu * retrotransposition in two neotropical primate genera. Moreover, identification of novel *MECP2* transcripts in *Callimico* suggests that part of a homologous human intronic region might be expressed, and that the potential open reading frame in this region might be a subject of interest in RTT patients who carry an apparently normal *MECP2* sequence.

**Electronic supplementary material:**

The online version of this article (doi:10.1186/s12863-015-0240-x) contains supplementary material, which is available to authorized users.

## Background

A serious clinical entity with arrested development between 6 and 18 months of age, regression of acquired skills, loss of speech, stereotypical movements (classically of hands), microcephaly, seizures and mental retardation, as initially described by Rett [1], is presently known as Rett syndrome (RTT). Molecular studies later showed that association between the X-linked, methyl-CpG Binding Protein 2 gene (*MECP2)* involved in neural development and RTT accounts for the first known association between an epigenetic regulator and a human disease [2–4]. Loss of *MECP2* function, resulting from mutations in 85 to 90 % of individuals affected by RTT [2], is the primary cause of this syndrome, a dominant disorder occurring almost exclusively in females [2, 3].

*MECP2,* located on Xq28, is closely associated with the epigenetic mechanisms of DNA methylation and gene inactivation. MECP2, encoded by *MECP2*, is a chromatin-associated nuclear protein member of the methyl binding domain (MBD) family capable of binding a single methyl-CpG [5]. MECP2 is a multifunctional protein involved in nuclear organization, chromatin architecture, chromatin template binding, chromatin compaction and fiber binding, heterochromatin rearrangement [6], loop domain chromatin organization, pericentromeric heterochromatin structure, and DNA methylation [7]. MECP2 also plays roles in regulating splicing and mRNA template activity [8] and in activating and repressing transcription [6, 9], and is normally expressed in all tissues, but has a higher expression level in the brain during neuronal maturation [10]. However, in mice, MECP2 has not been detected in microglial cells from the retina, cortex, cerebellum and spinal cord, in intestinal epithelial cells, or in erythropoietic lineage cells, hair matrix keratinocytes, mature oocytes and spermatozoids [11].

In addition to its C-terminal and N-terminal regions, MECP2 consists of two functional domains, the methylated DNA binding domain (MBD) and the transcriptional binding domain (TRD), along with other relevant structures. MBD, the first of these domains, is required for recognition of and binding to methylated dinucleotides in the CpG islands of promoter regions; binding requires the presence of an A/T-rich sequence adjacent to the methylated CpG regions [12, 13]. TRD, the second domain, is specifically required for transcriptional silencing via recruitment of the chromatin remodeling co-repressor Sin3A and histone deacetylases [14]. Three other domains have also been identified: (i) a WW domain binding region involved in interactions with the WW regions of splicing factors [15]; (ii) an arginine-glycine repetitive region known to mediate RNA-protein interactions [16]; and (iii) two nuclear localization signals, one of which is inside the TRD.

**Fig. 1:**
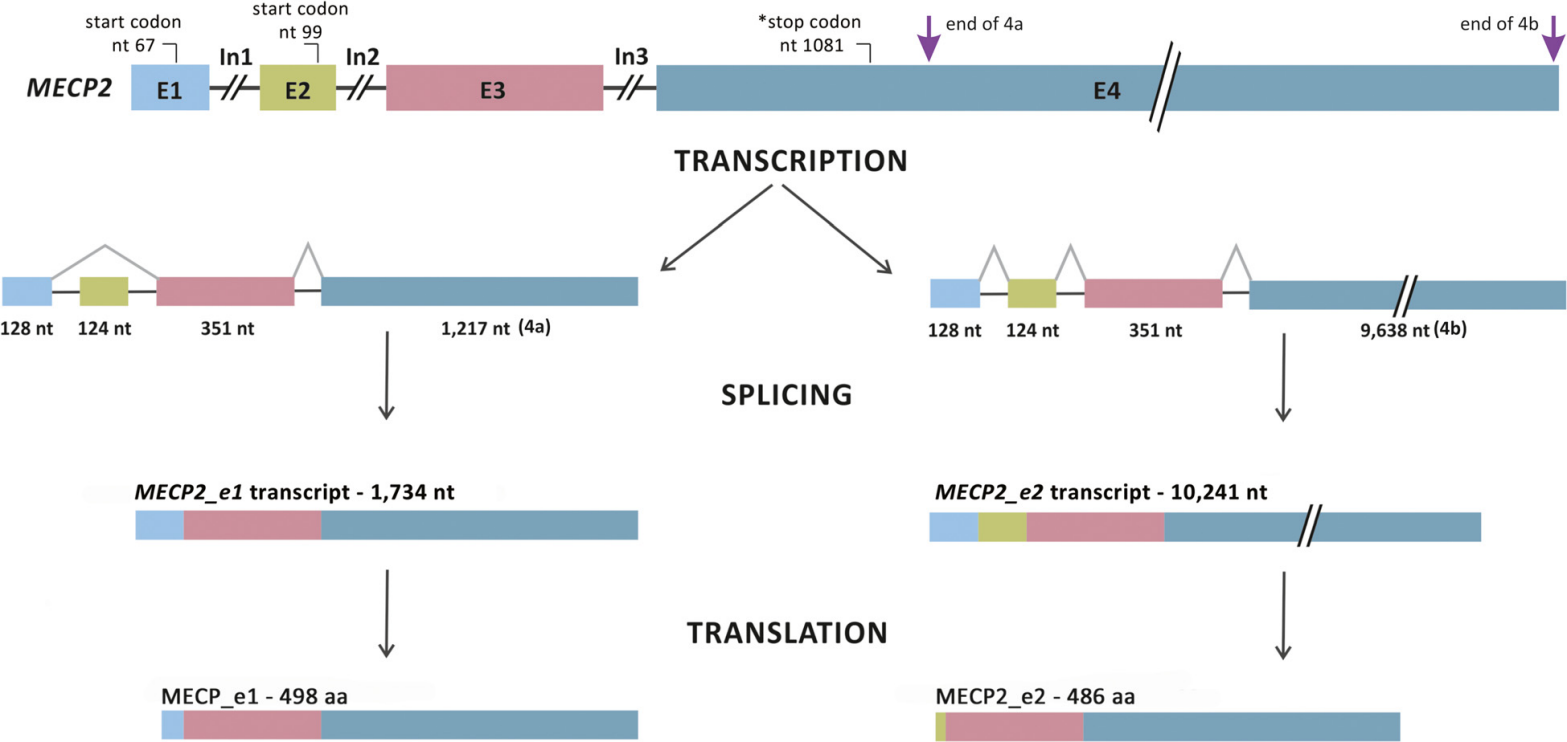
*MECP2* structure showing exons (E) and introns (In). The figure shows transcripts resulting from alternative splicing with 1,734 and 10,241 nucleotides (nt). Transcripts from different regions of exon 4 are indicated as 4a and 4b. Translation of mature mRNA molecules results in proteins of 498 and 486 amino acids (aa), MECP2_e1 and MECP2_e2, respectively

Through alternative splicing (Fig. 1), two mRNAs of different sizes are normally transcribed resulting in the following two isoforms: *MECP2_e1*, with a start codon in exon 1 and containing exon 1, 3 and 4 transcripts, and *MECP2_e2*, with a start codon in exon 2 and containing exon 1, 2, 3 and 4 transcripts. *MECP2_e1* encodes a protein of 498 amino acids and *MECP2_e2* a protein of 486 amino acids [17, 18]. *MECP2_e1* is the major isoform found in the brain and throughout development [4, 19, 20]. *MECP2_e1* has more relevance to the RTT phenotype [21], a finding also supported by studies on *MeCP2-e1* deficient mice that developed forelimb stereotypy, hindlimb clasping, excessive grooming and hypo-activity at 7 to 31 weeks prior to death [22]. Conversely, selective deletion of *MeCP2_e2* did not result in RTT-associated neurological phenotypes, but resulted in a survival disadvantage for embryos carrying a *MeCP2_e2* null allele of maternal origin. A specific requirement for MeCP2_e2 function was found in extraembryonic tissue, where selective loss of MeCP2_e2 resulted in placental defects [23].

Pathogenic and silent mutations, polymorphisms, and intronic variants have been identified in RTT patients [24, 25]. The most common mutation hotspots are reported to occur in the MBD and TRD domains and affect both MECP2 isoforms [18]. Additionally, the “RettBASE: IRSF *MECP2* Variation Database” (available at http://mecp2.chw.edu.au), records 862 different *MECP2* mutations. As seen in human males, lack of a functional MECP2 protein during embryonic development is fatal in early postnatal life [26]. *In vivo* experiments with transgenic mice carrying the functional gene and a mutant allele with inducible expression showed that several characteristics of the RTT phenotype were retrievable in adult life after inducing expression of the mutant allele [27]. Nevertheless, *MECP2* duplication (*MECP2* duplication syndrome) also affects neural and motor development in humans, with similar characteristics to RTT displayed in male patients, while women with *MECP2* duplications exhibit normal cognitive abilities and the propensity for neuropsychiatric abnormalities (depression, anxiety, compulsions, and autism) [28].

Here, we investigated the *MECP2* gene from humans and other primates to determine the evolutionary divergence of its functional regions and to identify the nucleotide (nt) sites that might be under selective pressure. The molecular analyses revealed that *MECP2* is evolutionary conserved among the primates studied herein. We also report the presence of two independent *Alu* retrotranspositions in intron 3, and a new alternative exon that includes part of intron 2 in one neotropical (platyrrine) primate species.

## Methods

### DNA isolation, polymerase chain reaction (PCR) amplification and DNA sequencing

Blood and tissue samples were collected from 61 neotropical primates belonging to 16 genera and from *Pan troglodytes* (Additional file 1). The blood samples collected were obtained as part of a regular health checkup and disease control scheme for captive animals. Tissue samples were collected in the field or from dead animals (donated by Rio de Janeiro Zoo and São Paulo Zoo). All procedures followed the National Guidelines and Provisions of ICMBio (Instituto Chico Mendes de Biodiversidade Brazil; permanent license number 11375-1).

DNA was isolated from all the samples with the exception of *Callimico goeldii*. DNA quality was assessed by electrophoresis on 0.8 % agarose gels and quantification in a NanoDrop® 1.000 Spectrophotometer (Thermo Scientific, Waltham, MA, USA). Exons 2, 3 and 4 were amplified as reported previously [24]. Amplification of these regions allowed us to analyze in detail the MBD and TRD domains where most of the common mutation hotspots have been reported in humans [18]. To amplify a specific region of exon 4 a primer pair was designed (F = 5′-AAG GAG TCT TCT ATC CGA TCT GT-3′ and R = 5′-TGT CCA CAG GCT CCT CTC TG-3′) using PRIMER3 and *In silico PCR* (http://frodo.wi.mit.edu/primer3 and http://genome.ucsc.edu/cgi-bin/hgPcr, respectively). To analyze the noncoding regions we designed a primer pair to amplify intron 3 based on the genomic alignments from catarrhines and platyrrhines available in GenBank (Additional file 1): F = 5′-CAC GGA AGC TTA AGC AAA GG-3′ and R = 5′-CTG GGG ACT GTG AGG ACA AA-3′. PCR conditions were as follows: initial denaturation at 94 °C (2 min 30 s) followed by 35 cycles at 94 °C (30 s), 57 °C (45 s), and 72 °C (45 s) with a final elongation step at 72 °C (3 min). PCR products were purified using GFX™ PCR DNA and a Gel Band Purification kit® (Amersham Biosciences). Sequencing reactions were conducted with the same PCR primers and BigDye Terminator v3.1 Cycle Sequencing Kit® (Applied Biosystems) according to the manufacturer’s instructions. Reactions were run on an ABI3130/XL sequencing platform. Sequence data have been deposited in GenBank (see Additional file 1).

### RNA isolation and RNAseq

RNA was isolated from the mononuclear blood cells of *Brachyteles arachnoides* CPRJ2160, *C. goeldii* CPRJ2033 and *Sapajus robustus* CPRJ2456 using RNeasy® (Qiagen) and then quantified with a NanoDrop spectrophotometer (Thermo Scientific). RNA quality and integrity were checked by electrophoresis on 1 % agarose gels. Libraries were prepared with a TruSeq RNA Sample Prep Kit v2® (Illumina) following the protocol recommended by the manufacturer. Such libraries were subsequently tested by quantitative (q)PCR using the KK4824 Quantification Kit (KAPA) for validation. Clusters were generated for at least one lane of a PE Flowcell v.3 following the manufacturer’s protocol, and the 2 × 100 bp reads were analyzed with an Illumina HiSeq2500 platform. Cycles registered at least 80 % base calls with a Q30 quality score.

Data were converted to Fastq files by CASAVA v1.8.2 (Illumina), and the contigs were assembled using Trinity software [29] with default settings. Contigs over 200 bp were subjected to Basic Local Alignment Search Tool analysis against human *MECP2_e2* (NM_004992.3) using CLC Genomics Workbench 7 software (Qiagen). The reads were mapped using the contigs as the reference with Bowtie 2 [30], at an average coverage of 63X. Open reading frames were manually annotated with MEGA 5.1 [31]. Data have been deposited in GenBank (see Availability of Supporting Data).

### cDNA synthesis and reverse transcriptase (RT)-PCR

RNA from *B. arachnoides* CPRJ2160, *C. goeldii* CPRJ2033 and *S. robustus* CPRJ2456 was treated to remove DNA with an RQ1 RNAse-Free DNAse Kit® (Promega) and cDNA was subsequently synthesized with Superscript™ II Reverse Transcriptase® (Life Technologies). The constitutively expressed beta-2-microglobulin (*B2M*) gene was RT-PCR amplified to check the quality of the cDNA using Platinum *Taq* DNA polymerase buffer 1X (Invitrogen), 1.5 mM MgCl_2_, 0,25 mM of each dNTP, 1 U of Platinum *Taq* DNA polymerase (Invitrogen), 1 pmol of B2M-F 5′-ATG AGT ATG CCT GCC GTG TGA-3′ and 1 pmol of B2M-R 5′-CGG CAT CTT CAA ACC TCC ATG-3′ using the following conditions: 94 °C (2 min) followed by 35 cycles at 94 °C (30 s), 55 °C (30 s) and 72 °C (1 min).

A novel *C. goeldii* CPRJ2033 splicing product identified by RNAseq was validated and homologous contigs in *B. arachnoides* and *S. robustus* were searched using RT-PCR with the primers designed using PRIMER3 and *In Silico PCR* based on the *Callimico* data (F = 5′-TCG GAG AGA GGG CTG TGG-3′ and R2 = 5′-AAT TTT GTT GAG GAA AGA AGG CA-3′). PCR conditions were as follows: an initial denaturation at 94 °C (5 min) followed by 35 cycles at 94 °C (15 s), 65 °C (15 s), 72 °C (15 s) and a final elongation at 72 °C (15 min). PCR products were sliced from low melting-point agarose gels and sequenced.

### Molecular analyses

Three datasets were constructed, one from the *MECP2* exons coding for *MECP2_e2*, another from the exon 1 data, and a third one from the intron 3 sequences. Only one sequence was used when shared sequences were identified between individuals. When the sequences differed in length because of missing data, the larger sequence was used for analysis.

The *MECP2_e2* dataset contained 30 different sequences from 45 specimens (see Additional file 1). The *MECP2_e2* dataset included the RNAseq data for *B. arachnoides* CPRJ2160, *C. goeldii* CPRJ2033, and *S. robustus* CPRJ2456. The following additional data from GenBank were included: *Homo sapiens* (NG_007107), *Callithrix jacchus* (NC_013918), *Gorilla gorilla gorilla* (NC_018447), *Macaca fascicularis* (NC_022292), *Macaca mulatta* (NW_001218202), *Nomascus leucogenys* (NC_019841), *Pan paniscus* (NW_003869946), *P. troglodytes* (NC_006491), *Papio anubis* (NC_018172), *Pongo abelii* (NC_012614), *Saimiri boliviensis* (NW_003943798) and *Otolemur garnettii* (NW_003852644). For *P. abelii*, the recommendation described in XM_009235414.1, that is, “the sequence of the model RefSeq transcript was modified relative to its source genomic sequence to represent the inferred CDS: deleted 1 base in 1 codon”, was followed.

The exon 1 dataset comprised 15 sequences and included data from GenBank and RNAseq from the three species studied herein (Additional file 2), while the intron 3 dataset comprised 44 different sequences from 70 specimens (Additional file 3). All the datasets were aligned with Mega 5 [31]. Amino acid sequences were deduced from the aligned data and were compared with the human reference sequence (NP_004983). Amino acid substitutions were analyzed with respect to the human mutations reported in RettBASE: IRSF *MECP2* Variation Database (http://mecp2.chw.edu.au). Nucleotide sequences encoding *MECP2_e2* were analyzed to identify codons under negative, neutral or positive selection through use of SLAC, FEL, REL, PARRIS and MEME (www.datamonkey.org); [32] and using a phylogenetic topology reported previously [33].

The *MECP2* nt sequences of intron 3 (excluding gaps and *Alu* inserts) were used for phylogenetic reconstruction and for inferring the best model of evolution, as based on ModelGenerator v. 0.85 [34]. Kimura’s K80 model with gamma distributed rate heterogeneity was used for phylogenetic reconstructions with PHYML 3.0 [35] for maximum likelihood (ML), and with MrBayes 3.2.1 [36] for Bayesian analysis. In MrBayes, the Markov chain Monte Carlo algorithm was implemented using two independent runs with four chains each and the cold chains were sampled every 100th generation until 10,000 trees were obtained (with a burn-in of 1,000). Support for each node in the ML topology was estimated by aLRT [37], and by bootstrap analyses based on 1,000 replicates.

*Alu* elements were detected in *Cebus*, *Sapajus* and *Nomascus* capuchin genera by manual alignment and specific *Alu* families were identified with RepeatMasker (http://repeatmasker.org). *Cebus* and *Sapajus* consensus sequences, estimated using BioEdit [38], were compared with the *N. leucogenys* sequence using the *Alu* data available in RepBase (http://www.girinst.org).

## Results and discussion

### Amino acid substitution comparisons

**Fig. 2:**
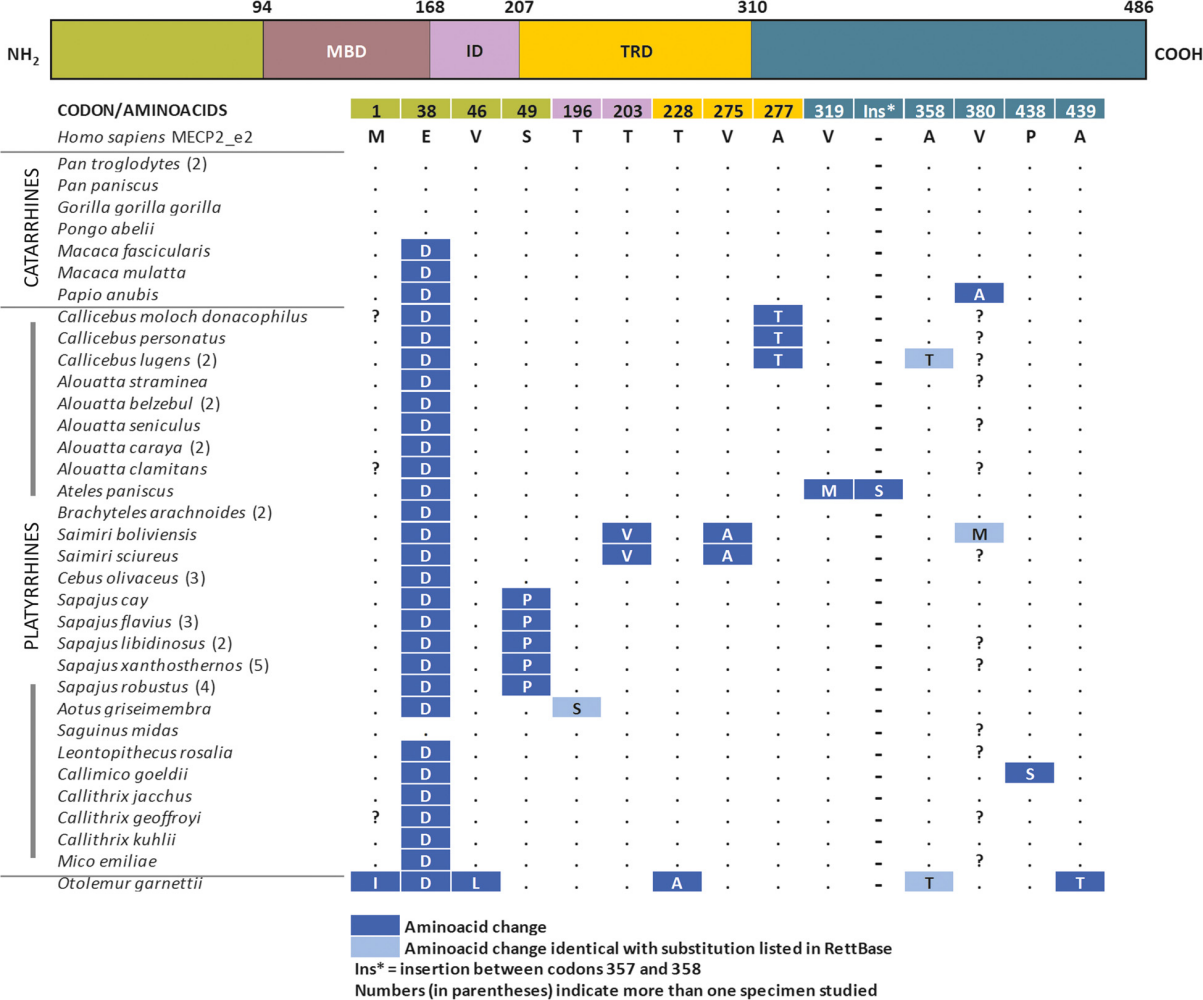
Comparison of human MECP2_e2 amino acid sequences with those from other primates. Top bar indicates different MECP2 regions with codon numbers delimiting each region. ID = interdomain. Numbers inside parentheses indicate the number of specimens analyzed. Ins* indicates an insertion between codons 357 and 358

Comparisons of the *MECP2_e2* coding region of nonhuman primates revealed amino acid substitutions in 14 codons when compared with the human sequence (Fig. 2), and one in-frame insertion of a single serine codon, between codons 357 and 358 in *A. paniscus*, was identified. In terms of amino acid polarity, analysis of the substitutions identified three that occurred between nonpolar residues, followed by polar for nonpolar substitutions (*n* = 2), nonpolar for polar substitutions (*n* = 3), one negatively charged for another negatively charged substitution and substitution of one polar residue for another polar residue. We observed three different substitutions in three codons of the N-terminal region of *MECP2_e2*. Of these, E38D (substitution of a negatively charged residue by another negatively charged residue) occurred in all of the non-hominid primates. The exception to this was the *Saguinus midas* sequence, which shared the same residue to that of the human sequence. Our interpretation of these findings lends to the proposal that aspartate was the ancestral residue of codon 38 and that a D38E substitution must have occurred twice and independently in the phylogenetically distant lineages leading to the large hominoids and *Saguinus*. Another substitution was found in the N-terminal region of MECP2_e2 (S49P, which involves a nonpolar for a polar substitution); this was restricted to the *Sapajus* genus and was absent in all other taxa studied herein, including *Cebus*, a closely related genus. Two substitutions, T203V (in the interdomain) and V275A (in the TRD domain), were restricted to two species of *Saimiri,* and A277T (in the TRD domain) to *Callicebus* species. Three different substitutions were found in four species; these were in the interdomains or C-terminal regions (T196S, A358T and V380M) previously listed in RettBASE as being nonpathogenic in humans. Indeed, T196S and A358T are polymorphic variants and V380M is a variant with unknown effects. Finally, the *O. garnettii* alignment showed an isoleucine codon that matched the first methionine codon in the majority of the other primates, and that the first methionine codon in this species corresponded to codon five of human *MECP2_e2*.

Amino acid substitutions did not occur in the MBD domain, a region of critical relevance in that it binds exclusively to DNA that contains one or more symmetrically methylated CpGs in promoter regions [12, 13]. In contrast, three amino acid substitutions were found in the TRD domain (T228A, V275A, and A277T) but these did not occur in the sites previously associated with RTT [39].

### Identification of sites under selection

Our findings suggested that *MECP2* is highly conserved in primates. Few amino acid substitutions occurred twice and independently according to the phylogenetic relationships of the primate lineages [33]. To evaluate whether specific amino acid residues were under positive or negative selection an analysis was carried out using Datamonkey [32]. Three codons (38, 358 and 380) were found to be under positive selection by REL only, the least stringent algorithm of the three we used for analyzing selection (Table 1). None of these sites occurred in critical regions. Indeed, residue 38 occurred in the N-terminal region, while two other, 358 and 380, occurred in the C-terminal region. Conversely, a total of 48 sites were found to be under negative selection in the different regions, 23 of which were found consistently by REL, FEL and SLAC (Table 1). Five of the sites were found in the N-terminal region, one in the MBD region, 10 in the TRD region, and seven in the C-terminal region. Furthermore, no evidence of positive selection on any codon was apparent by PARRIS analysis. These findings highlight the high evolutionary conservation in *MECP2_e2* although the possibility that the restricted, interspecific variations might affect posttranslational modification of MECP2 cannot be ruled out.

**Table 1:** Codons under selective pressure as inferred by algorithms of FEL, REL and SLAC analysis

FEL analysis		REL analysis		SLAC analysis	
Codon under selection	Normalized dN-dS (*p*-value)	Codon under selection	Normalized E [dN-dS] (posterior probability)	Codon under selection	dN-dS (*p*-value)
24	-54.388 (0.015)				
26	-64.692 (0.001)	26	-5.640 (0.999)		
28	-23.921 (0.015)				
		38*	-0.001 (0.834)		
45	-25.058 (0.037)				
48	-28.501 (0.029)				
50	-116.021 (0.001)	50	-8.259 (0.999)	50	-3.401 (0.037)
54	-74.389 (0.001)	54	-8.845 (1)	54	-5.668 (0.004)
62	-171.052 (0.000)	62	-11.116 (1	62	-6.802 (0.001)
64	-53.205 (0.006)	64	-4.313 (0.999)		
68	-121.797 (0.000)	68	-10.816 (1)	68	-5.668 (0.005)
69	-25.298 (0.046)				
70	-22.768 (0.024)				
72	-35.866 (0.020)				
75	-61.881 (0.002)	75	-6.324 (0.999)	75	-3.401 (0.037)
81	-43.081 (0.021)				
100	-19.480 (0.045)				
107	-95.287 (0.002)	107	-8.043 (0.999)	107	-3.944 (0.025)
109	-60.174 (0.011)	109	-4.356 (0.999)		
140	-35.109 (0.022)				
201	-40.376 (0.017)				
216	-23.921 (0.042)				
221	-44.476 (0.002)	221	-3.753 (0.999)	221	-4.380 (0.014)
229	-120.451 (0.000)	229	-10.822 (1)	229	-5.668 (0.004)
251	-46.877 (0.005)	251	-4.290 (0.999)	251	-4.535 (0.012)
252	-37.440 (0.025)				
257	-203.416 (0.000)	257	-11.114 (1)	257	-9.469 (0.000)
259	-39.143 (0.012)	259	-3.068 (0.999)	259	-3.401 (0.037)
272	-202.211 (0.000)	272	-11.134 (1)	272	-6.802 (0.001)
278	-36.279 (0.013)	278	-2.750 (0.999)	278	-3.401 (0.037)
280	-43.065 (0.009)	280	-3.467 (0.999)	280	-3.401 (0.037)
281	-52.084 (0.006)	281	-4.529 (0.999)	281	-3.401 (0.037)
311	-92.609 (0.000)	311	-9.538 (1)	311	-4.535 (0.012)
314	-90.671 (0.000)	314	-9.087 (0.9999)	314	-5.034 (0.008)
327	-41.076 (0.009)	327	-3.273 (0.999)	327	-4.466 (0.016)
328	-38.107 (0.005)	328	-2.958 (0.999)	328	-3.401 (0.037)
332	-29.179 (0.029)				
342	-86.299 (0.000)	342	-9.663 (1)	342	-5.668 (0.004)
		359*	0.085 (0.875)		
361	-40.741 (0.009)				
380	-96.464 (0.002)	380	-8.703 (0.999)	380	-6.060 (0.012)
		381 *	-0.078 (0.797)		
382	-113.953 (0.001)	382	-9.995 (1)	382	-7.575 (0.004)
				390	-4.545 (0.037)
				392	-4.545 (0.037)
394	-32.544 (0.041)				
420	-37.880 (0.029)				
425	-24.576 (0.032)				
428	-54.374 (0.010)	428	-4.557 (0.999)	428	-4.190 (0.019)
441	-21.464 (0.028)				
443	-23.477 (0.038)				

Marked sites with asterisk are under positive selection. Unmarked sites are under negative selection. PARRIS and MEME did not show evidence of positive selection

### Analysis of the exon 1 dataset

Exon 1 could not be amplified, a result probably caused by the high number of repetitive motifs at the 5′-UTR and intron 1 of the human *MECP2* sequence (GenBank NG007107) and in other primate sequences with available data. For some primates, including *P. paniscus* (NW003869946), *S. boliviensis* (NW003943798), and *Rhinopithecus roxellana* (NW010801008), the complete genomic data do not contain information on *MECP2* exon 1 and its flanking regions, confirming the difficulties involved in sequencing this genome region. However, *Callimico*, *Sapajus* and *Brachyteles* RNAseq data allowed for identification of exon 1 in these genera. Comparisons of exon 1 revealed remarkable evolutionary conservation and phylogenetic signal grouping the three cebids (*Callithrix, Callimico* and *Sapajus*). These genera also shared a codon deletion in a poly-alanine region. The lack of critical information on exon 1 for the majority of the species we studied stopped us from conducting a more comprehensive evolutionary analysis of a region of critical relevance in RTT. Further approaches, via whole genome sequencing and RNAseq analysis might provide the relevant information.

### Phylogenetic analysis based on intron 3

Taking into account the limited sequence divergence of the *MECP2* coding regions in the species analyzed herein, and the dearth of sequence data for several taxa, a phylogenetic analysis based on intron 3 was carried out to evaluate whether *MECP2* evolution was in agreement with a previous phylogenetic proposal [33] based on a large dataset (~34,000 bp of aligned sequences) of autosomal genes and genes allocated to X and Y chromosomes. In total, 44 different sequences with a similar length to the human intronic region (*ca.* 756 bp) were found, except for all *Sapajus* and *Cebus* specimens in which we identified an *Alu* insert (in nt 418 or 419 of intron 3, respectively) of approximately 300 bp in length. Regardless of this insert, the majority of the intron 3 sequences were species-specific except for some sequences that were shared between some *Sapajus*, *Callithrix* and *Leontopithecus* species.

**Fig. 3:**
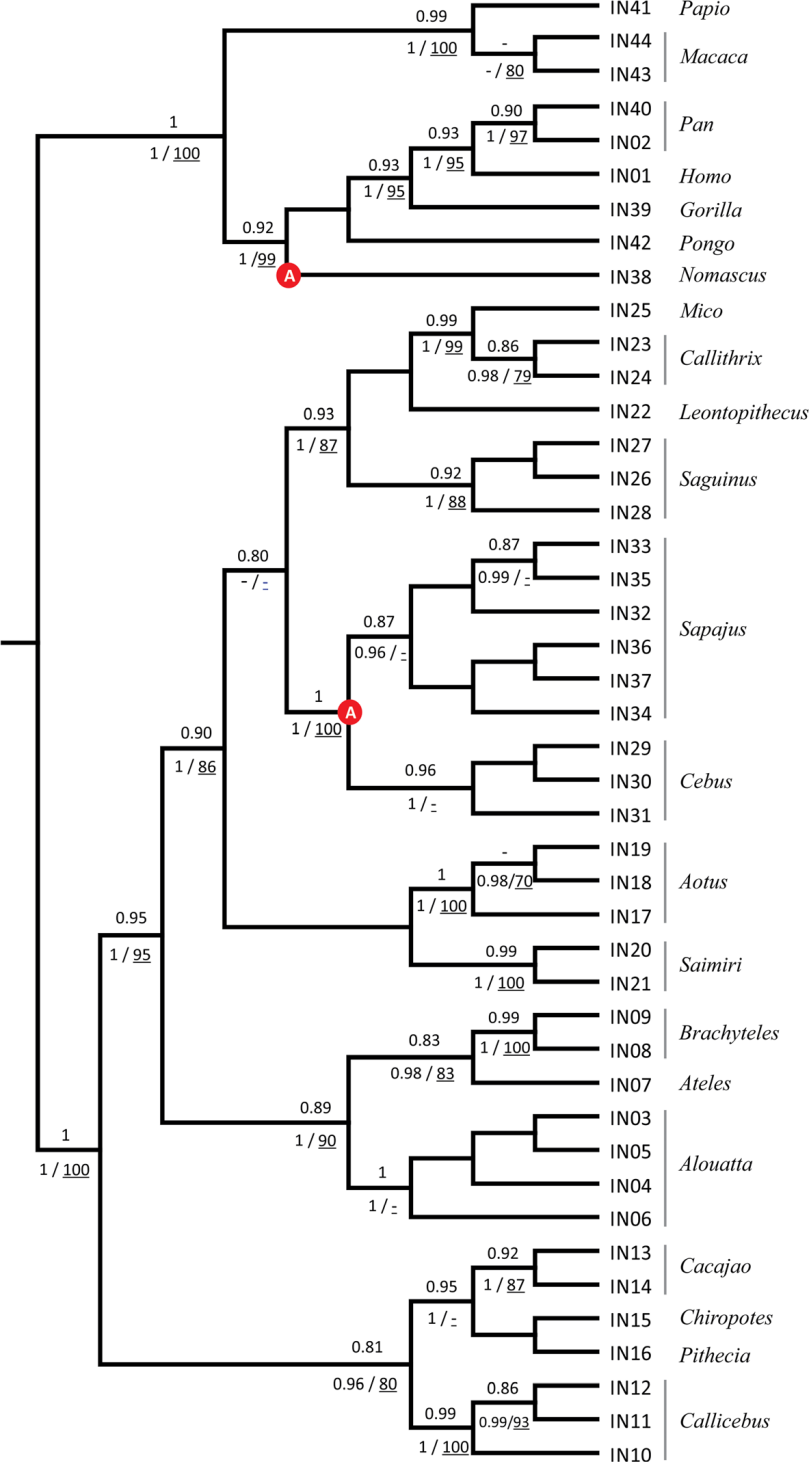
Maximum likelihood topology based on *MECP2* intron 3. Values above the nodes indicate the aLRT support estimates. Values below nodes indicate the posterior probabilities of the Bayesian tree and bootstrap estimates. Red circles indicate *Alu* insertions

ML and Bayesian analyses of intron 3 generated very similar topologies to the proposed primate phylogeny [33], with three evolutionary lineages corresponding to the main families of the neotropical primates (Cebidae, Atelidae and Pitheciidae). The ML topology (Fig. 3), however, showed a different arrangement within the Cebidae, viz. (((*Sapajus*,*Cebus*)Callitrichini)(*Aotus*,*Saimiri*)), and within the Pitheciidae ((*Pithecia,Chiropotes*)*Cacajao*), although these discordant arrangements were supported by low bootstrap and aLRT estimates. The lack of a consensus arrangement for the Cebidae primates probably results from the short time span between their origin and the radiation of their derived lineages [33, 40, 41].

### Independent *Alu* insertions

The intron 3 sequence alignments of neotropical species revealed the presence of an *Alu* insert shared by species of two closely related genera: *Cebus* and *Sapajus*. This insert was not found in any other primate except for a similar, albeit inverted *Alu* insert in nt 437 of intron 3 from *N. leucogenys* (NC_019841). RepeatMask analysis showed that the *Cebus/Sapajus* insert shared high similarity with the *Alu* subfamily S, while the *N. leucogenys* insert was found to be similar to the *Alu* Y subfamily (Additional file 3). These findings indicate that these insertions occurred twice and independently. The *Alu* insertion must have occurred in the common stock of *Cebus* and *Sapajus*, confirming the monophyly of these taxa [42]. The subsequent divergence of *Alu* inserts, resulting in *Cebus*-specific and *Sapajus*-specific lineages, points to their separation as is also evident by the presence of a derived S49P substitution in *Sapajus*.

### Expression of *MECP2* transcripts in blood cells

Because alternative splicing of *MECP2* results in different protein isoforms and in view of their importance to normal development in humans, coverage of mRNAs coding for isoforms 1 and 2 was analyzed by RNAseq *de novo* assembly in the three platyrrhine species (*S. robustus*, *B. arachnoides*, and *C. goeldii*). Homologous contigs for each human *MECP2* isoform were identified, while a third novel *C. goeldii* CPRJ2033 contig of 525 nt was also identified. This contig is homologous to a region that includes human exons 1 and 2 and a 270 bp region corresponding to a noncontiguous region of human intron 2 (from nt 12,518 to 12,788). RNAseq analysis showed a higher coverage of *MECP2_e2* mRNA than *MECP2_e1* in *S. robustus* and *C. goeldii*, with a *MECP2_e2*:*MECP2_e1* ratio equal to 1.20 and 1.88 respectively. Conversely, in *B. arachnoides*, the ratio was 0.76. Differences in sequence coverage might reflect differences in expression or, alternatively, technical artifacts reflecting the efficiency of amplification of the different isoforms caused by the GC-rich composition of exon 1 and its flanking regions (Additional file 4).

Regardless of these findings, differential expression of MECP2 isoforms might also reflect the different epigenetic changes that can vary between nonhuman primate species. Some evidence has been reported supporting the role of methylation in RTT [43], in the expression of MeCP2 isoforms in different brain regions [20], and in differentiating neural stem cells [44]. Further studies should help to clarify the role of epigenetics in regulating expression of MECP2 isoforms in different species.

**Fig. 4:**
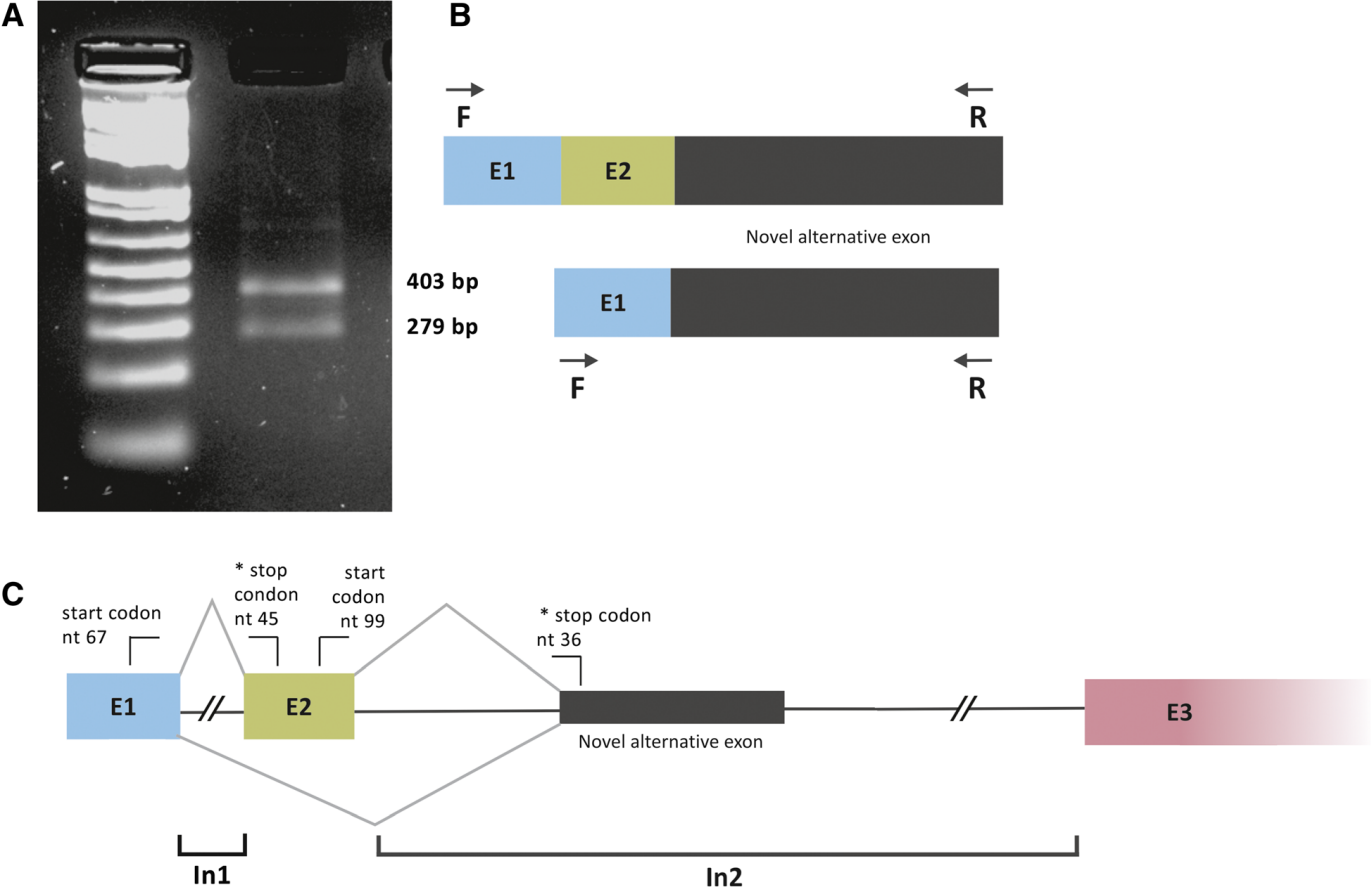
RT-PCR products corresponding to novel transcripts in *Callimico goeldii*. **a** RT-PCR products following amplification of a 525 bp contig resulted in two fragments of 403 and 279 bp. **b** Transcript composition (E1 = exon 1; E2 = exon 2). Arrows indicate the primer positions. The new alternative exon, homologous to part of human intron 2, is shown in black. **c** Schematic representation of the novel exon and the alternative splicing resulting in the two new transcripts. Start and stop codons indicate three likely open reading frames. IN1 = intron 1; IN2 = intron 2; IN3 = intron 3; E1 = exon 1; E2 = exon 2; E3 = exon 3

RT-PCR confirmed the presence of a third novel transcript of 525 nt. From this, there were two amplified products, one fragment of the expected size (403 bp) and an additional 279 bp product that was *C. goeldii*–specific*.* The larger product matched the 525 bp contig while the smaller one matched exon 1 and the noncontiguous fragment of intron 2 (Fig. 4).

Sequence analysis of the RT-PCR products showed that, excluding the primers, the 403 bp fragment contained part of exon 1 (76 bp), the complete exon 2 (124 bp) and part of the noncontiguous segment of intron 2 (162 bp) confirming the presence of the exon2–intron2 segment junction found by the RNAseq *de novo* assembly. The deduced amino acid sequence of this fragment, from the ATG initiation codon of exon 1 used for translation of human *MECP2_e1* to an in-frame stop codon inside exon 2, corresponded to a 33 amino acid polypeptide. Alternatively, the possibility exists that the 403 bp fragment could produce a 26 amino acid polypeptide following translation from the ATG initiation codon at exon 2, and this polypeptide might be used for translation of human *MECP2_e2* to an in-frame stop codon inside intron 2. The smaller 279 bp fragment contained the same part of exon 1 and the same part of the noncontiguous segment of intron 2. A sequence of 37 amino acids was deduced from the ATG initiation codon of exon 1 used for translation of human *MECP2_e1* to the same in-frame stop codon inside intron 2.

Identification of a transcribed region in *Callimico*, which is homologous to part of human exon 2 and part of intron 2, points to evolutionary conservation of these regions regardless of their expression patterns. Why this region is transcribed in *Callimico* is an open question. Human intron 2 is not analyzed in conventional RTT genetic testing and many RTT patients appear to have a normal *MECP2* sequence. Expression of this region in humans, where potentially deleterious mutations are expressed, might explain this paradox. Whole genomic analysis of *Callimico*, which is presently under way, will allow identification of the boundaries surrounding the new alternative exon and comparisons of splice donor/acceptor sites in humans. Future *in silico* analysis may provide molecular explanations for the novel exon, such as whether it is related to novel exonic splicing enhancers or splice donor/acceptor signals.

## Conclusions

These studies show that despite the remarkable evolutionary conservation of *MECP2_e2*, one in-frame codon insertion occurred in *Ateles paniscus*, and one region of intron 3 was affected by trans-specific retrotransposition  (*Alu *insertion). Moreover, identification of novel *MECP2* transcripts in *Callimico* suggests that part of the homologous intronic region in humans might be expressed, and that the potential open reading frame in this region might be a subject of interest in RTT patients who carry an apparently normal *MECP2* sequence. This is particularly relevant because alterations in this gene are associated with RTT in humans. Here, we found that evolutionary variation in *MECP2* is not correlated with any pathological alteration previously associated with RTT.

### Availability of supporting data

The following sequence data have been deposited in GenBank: *MECP2* intron 3 (accession numbers KM206798 to KM206856), *MECP2* exon 2, 3 and 4 (accession numbers KM206857 to KM207008), and *MECP2* isoforms (accession numbers KR265897 to KR265903). Accession numbers of intron and exons are listed for each individual sample in Additional file 1. Intron 3 data and topology have been deposited in TreeBASE (Study Accession URL: http://purl.org/phylo/treebase/phylows/study/TB2:S17761) .

## Additional files

Additional file 1:
**List of specimens, field numbers and GenBank accession numbers of sequence data.**
Additional file 2:
**Alignment of the available data for exon 1.**
Additional file 3:
**Alignment of** ***Alu*** **sequences.**
Additional file 4:
**Transcript coverage following** ***de novo*** **assembly of** ***MECP2*** **in** ***Sapajus*** **,** ***Brachyteles*** **and** ***Callimico*** **.** The number on the left side of each illustration indicates the highest level of coverage.
