# Supplementary material for: MECP2, a gene associated with Rett syndrome in humans, shows conserved coding regions, independent Alu insertions, and a novel transcript across primate evolution
Source: BMC Genet. 2015 Jul 7;16:77. doi: 10.1186/s12863-015-0240-x (PMC4493987; doi:10.1186/s12863-015-0240-x)

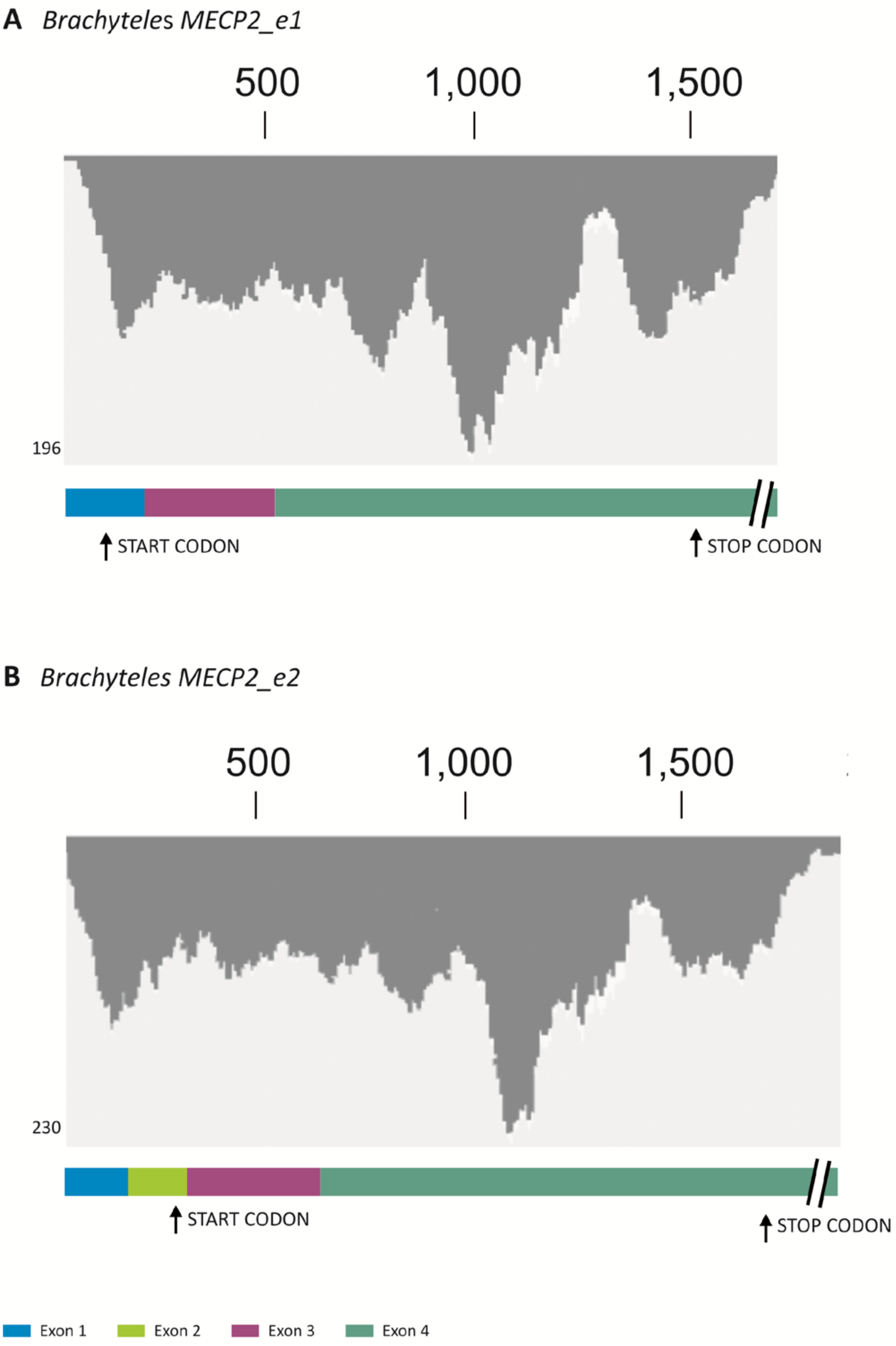
A Brachyteles MECP2_e1
500
1,000
1,500
196
START CODON
STOP CODON
B Brachyteles MECP2_e2
500
1,000
1,500
230
START CODON
STOP CODON
Exon 1
Exon 2
Exon 3
Exon 4

A *Sapajus MECP2_e1*

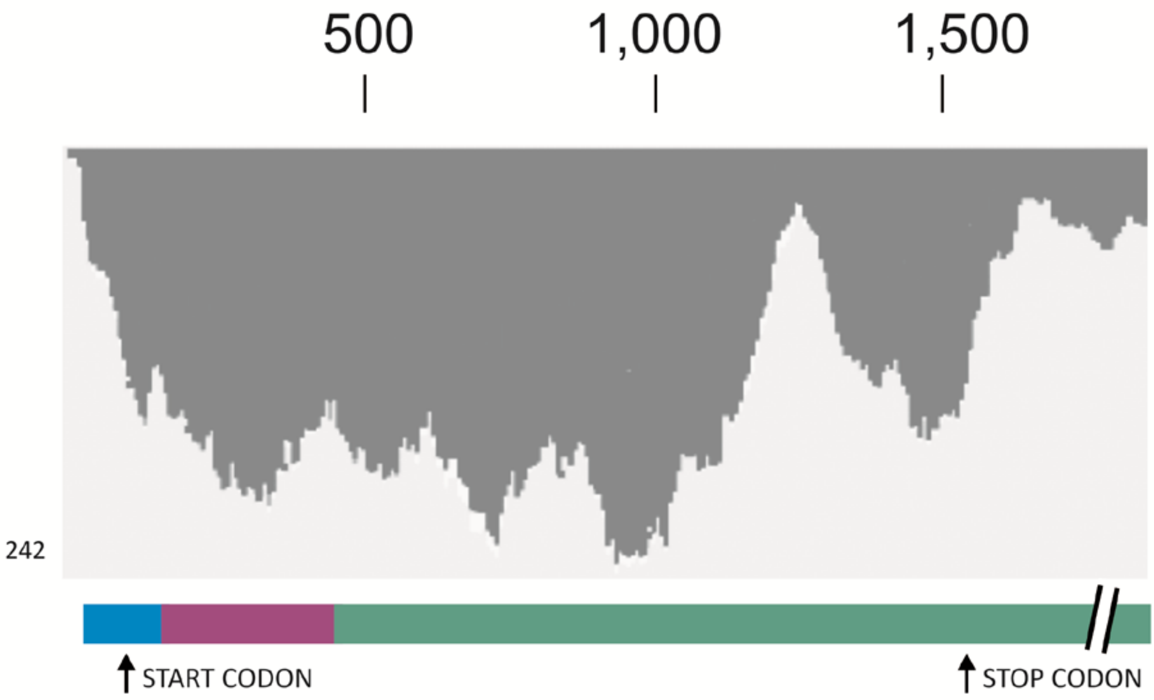

B *Sapajus MECP2_e2*

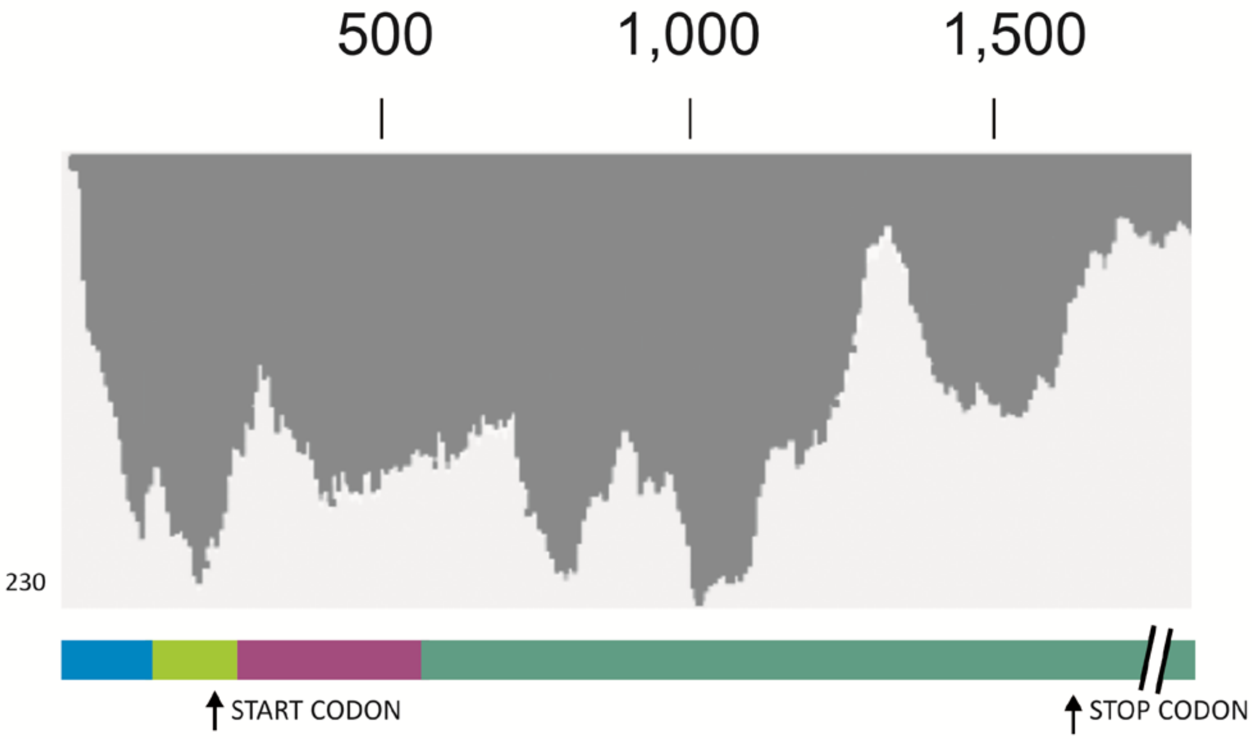

Exon 1 Exon 2 Exon 3 Exon 4

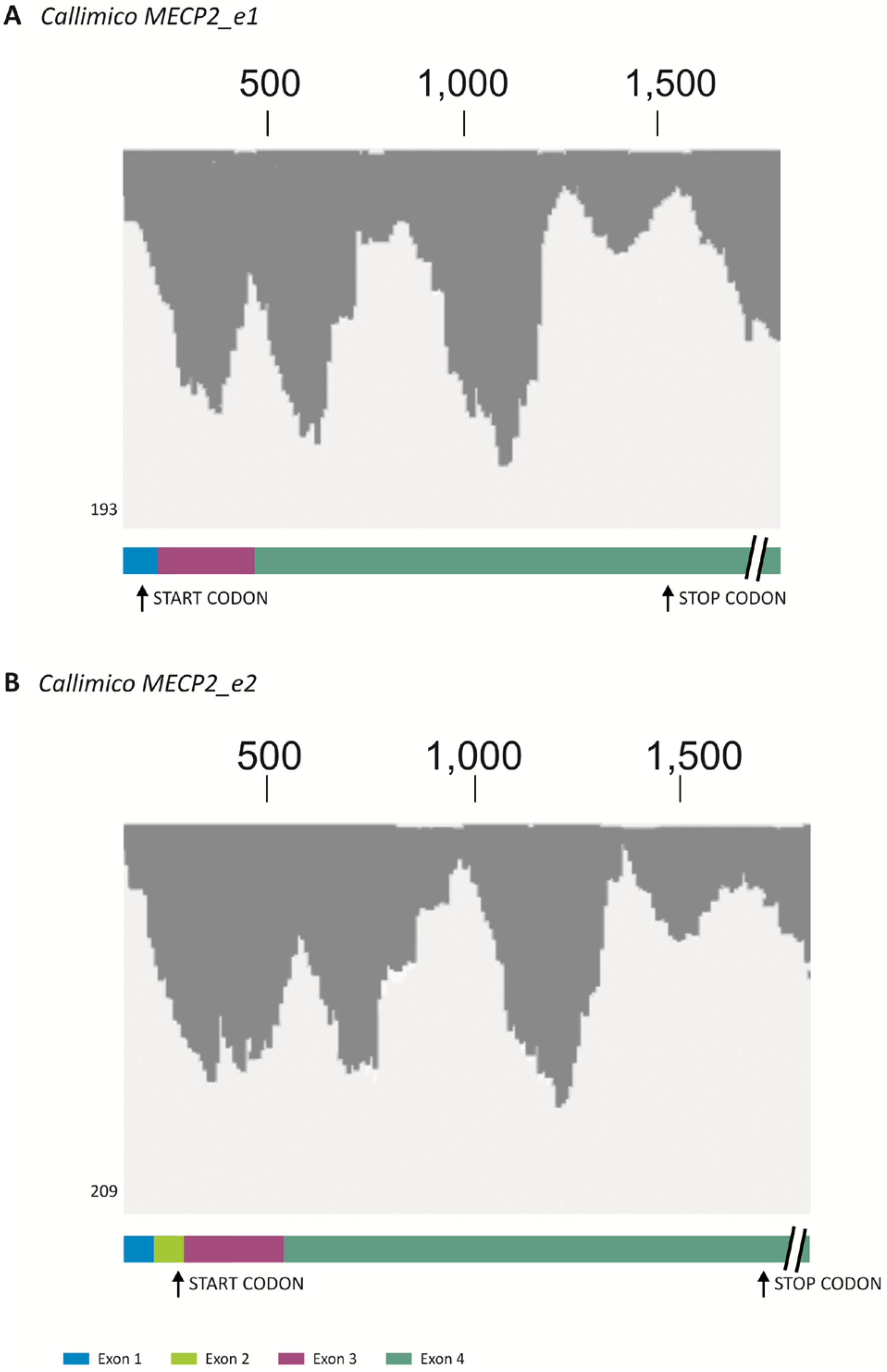
A Callimico MECP2_e1
500
1,000
1,500
193
START CODON
STOP CODON
B Callimico MECP2_e2
500
1,000
1,500
209
START CODON
STOP CODON
Exon 1
Exon 2
Exon 3
Exon 4

Callimico MECP2 new isoform

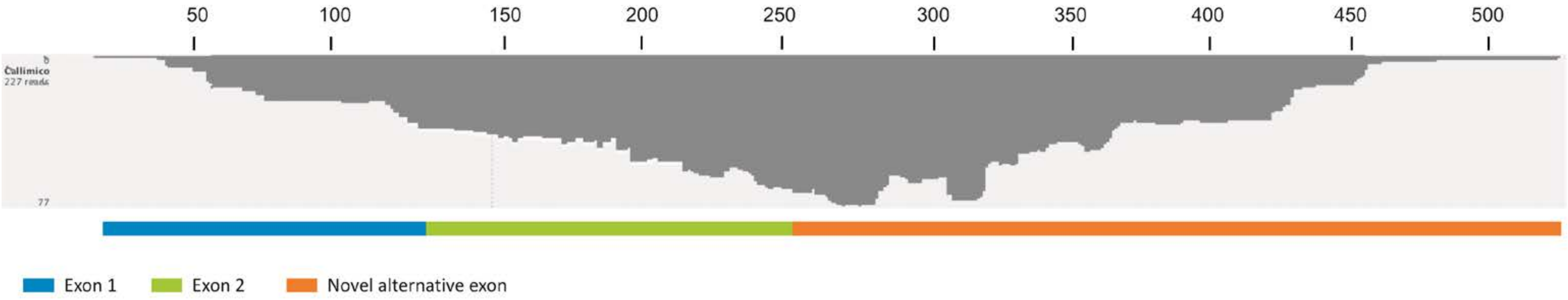

Supplement: Additional file 4: — Transcript coverage following de novo assembly of MECP2 in Sapajus , Brachyteles and Callimico . The number on the left side of each illustration indicates the highest level of coverage. [file 12863_2015_240_MOESM4_ESM.pdf]
